# Short-term cost analysis of raltegravir versus atazanavir + ritonavir or darunavir + ritonavir for treatment-naive adults with HIV-1 infection in the United States

**DOI:** 10.1371/journal.pone.0203293

**Published:** 2018-08-30

**Authors:** Anita J. Brogan, Ashley E. Davis, Bridgett Goodwin

**Affiliations:** 1 RTI Health Solutions, Research Triangle Park, NC, United States of America; 2 Merck & Co., Inc., Kenilworth, NJ, United States of America; Azienda Ospedaliera Universitaria di Perugia, ITALY

## Abstract

The head-to-head AIDS Clinical Trial Group (ACTG) 5257 clinical trial found raltegravir (RAL) to be superior to atazanavir + ritonavir (ATV/r) and darunavir + ritonavir (DRV/r), when used in combination with emtricitabine/tenofovir disoproxil fumarate (FTC/TDF) by treatment-naive adults with HIV-1 infection, in a 96-week composite endpoint combining virologic efficacy and tolerability. The objective of this study was to estimate the total HIV treatment costs associated with these three regimens in the United States.

**Methods:**

Ninety-six–week costs for antiretroviral drugs, adverse event management, and HIV care for individuals initiating RAL, ATV/r, or DRV/r as first-line therapy for HIV-1 infection were estimated using an economic model. Efficacy and safety data (mean CD4 cell count changes, discontinuation rates, grade 3/4 adverse event incidence) for each regimen through 96 weeks of treatment were taken from the ACTG 5257 clinical trial. Antiretroviral drug costs for each initial regimen and for each substitution regimen, as used by individuals who discontinued their initial regimen, were based on wholesale acquisition costs. Adverse event management costs and HIV care costs, stratified by CD4 cell count range, were taken from published sources and inflated to 2016 dollars. Scenario and sensitivity analyses were conducted to assess the robustness of the results. Cost outcomes were discounted at an annual rate of 3.0%.

**Results:**

Total 96-week costs were $81,231 for RAL, $88,064 for ATV/r, and $87,680 for DRV/r, where differences were primarily due to lower antiretroviral drug costs for RAL than for ATV/r or DRV/r. These results were found to be robust in scenario and sensitivity analyses.

**Conclusions:**

Relative to the DRV/r and ATV/r regimens, the RAL regimen had the lowest cost for treatment-naive adults with HIV-1 infection in the United States.

## Introduction

As of 2013, an estimated 1,242,000 people were living with HIV-1 infection in the United States (US) [1]. While improvements in the treatment of HIV-1 infection during the last decade have increased life expectancy and quality of life for individuals with HIV, the cost of care has risen. The first HIV regimen chosen for a treatment-naive individual has implications for treatment and care in the long-term. When considering the available treatments, it is important to select the most effective, safe, and economical regimen that meets the patient's individual needs.

The AIDS Clinical Trial Group (ACTG) 5257 head-to-head clinical trial investigated common integrase and protease inhibitors for treatment-naive individuals not receiving efavirenz or other nonnucleoside reverse transcriptase inhibitors. The objective of this large study (N = 1,809) was to compare the efficacy and tolerability of raltegravir (RAL) 400 mg twice daily, atazanavir + ritonavir (ATV/r) 300 mg/100 mg once daily, and darunavir + ritonavir (DRV/r) 800 mg/100 mg once daily, each in combination with emtricitabine/tenofovir disoproxil fumarate (FTC/TDF) 200 mg/300 mg once daily, among treatment-naive adults in the US [2, 3]. The results of the ACTG 5257 trial indicated that, at 96 weeks of follow-up, the RAL regimen was superior to the ATV/r and DRV/r regimens in a composite endpoint combining virologic efficacy and tolerability [2, 3].

However, ACTG 5257 did not investigate the economic implications of these first-line HIV treatment regimens. Cost information is an essential complement to efficacy and safety data for prescribers and payers making decisions about HIV treatment in the US. The objective of this study was to estimate the total HIV treatment costs associated with the three antiretroviral (ARV) regimens examined in ACTG 5257—RAL, ATV/r, or DRV/r—when used in combination with FTC/TDF for treatment-naive adults with HIV-1 infection in the US.

## Materials and methods

### Model overview

Total 96-week costs associated with the three first-line ARV regimens examined in the ACTG 5257 clinical trial were estimated for the US setting using an economic model. The model followed a cohort of treatment-naive individuals with HIV-1 infection from treatment initiation through 96 weeks of treatment. Upon entering the model, individuals were assigned to a first-line ARV treatment arm: RAL, ATV/r, or DRV/r. After initiation of treatment, individuals could experience adverse events and changes in health status, indicated by health states based on CD4 cell count (< 50, 50–199, 200–349, 350–499, 500–649, 650–799, ≥ 800 cells/μL). Individuals who remained on first-line ARV treatment for all 96 weeks were considered successfully treated. Individuals who discontinued their first-line ARV regimen were reassigned to an alternative regimen, which they received for the remainder of the analysis.

**Fig 1:**
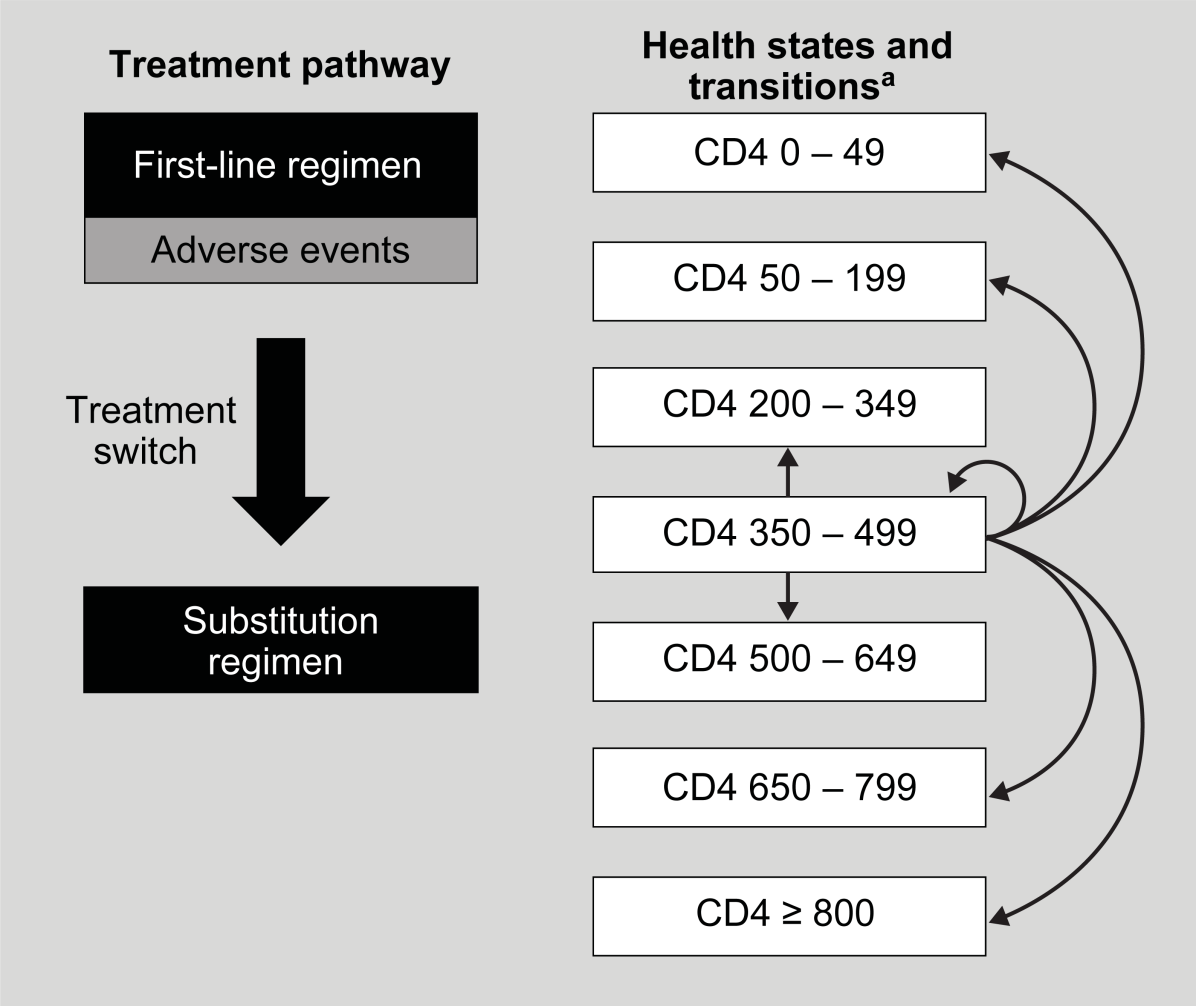
ACTG 5257 cost-analysis model overview. ^a^ As a simplification, transitions are shown for the 350–499 cells/**μ**L CD4 cell count health state; similar transitions are allowed from the other health states.

As individuals progressed through the model, they incurred costs for ARV drug treatment, adverse event management, and HIV care (i.e., disease monitoring and the treatment and prevention of opportunistic and other infections). For each treatment arm, per-person costs were estimated by category and in aggregate (Fig 1).

### Input parameter values

#### Modeled population

Baseline characteristics of the modeled cohort were based on the pooled ACTG 5257 intention-to-treat population (Table 1) [2]. This information was used to establish the distribution of the modeled cohort across the seven health states at treatment initiation.

**Table 1:** ACTG 5257 clinical trial baseline characteristics.

Characteristic	Pooled
N	1,809
Mean age (years)	37
Female	23.8%
HIV-1 RNA < 100,000 copies/mL	69.5%
Baseline CD4 cell count (cells/μL)^a^ [2]	
< 50	11.9%
50–199	17.7%
200–349	29.2%
350–499	27.7%
500–649	8.5%
650–799	3.6%
≥ 800	1.4%

ACTG, AIDS Clinical Trial Group; ATV/r, atazanavir + ritonavir; DRV/r, darunavir + ritonavir; RAL, raltegravir.

^a^ The pooled baseline characteristics for the intention-to-treat population from the ACTG 5257 clinical trial were used to establish the distribution of the modeled cohort across health states at treatment initiation. The intention-to-treat population received RAL (n = 603), ATV/r (n = 605), or DRV/r (n = 601).

#### Clinical inputs: Efficacy and tolerability

**Fig 2:**
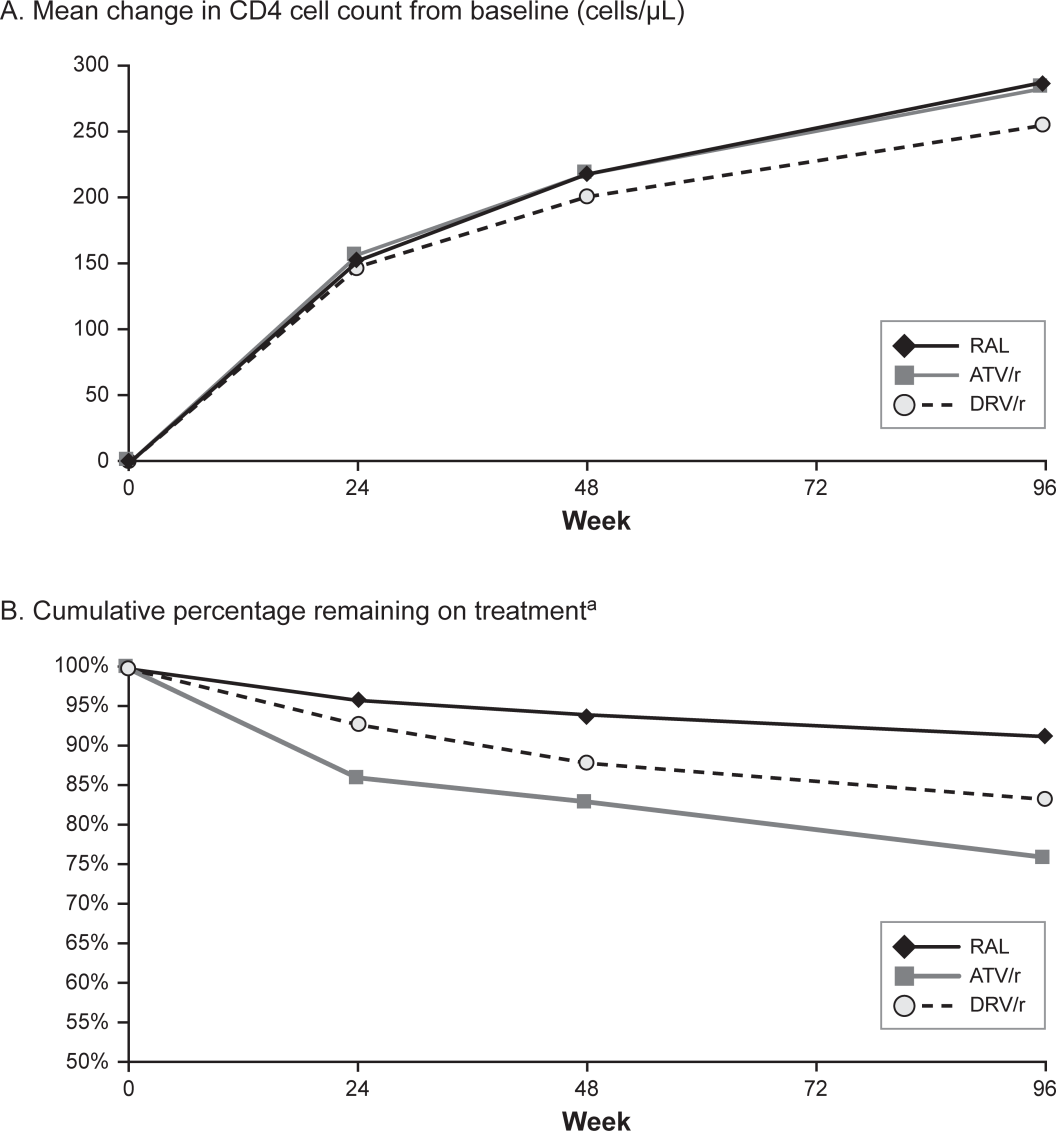
ACTG 5257 clinical trial efficacy and tolerability data. **(A) Mean change in CD4 cell count from baseline (cells/μL). (B) Cumulative percentage remaining on treatment.** ACTG, AIDS Clinical Trial Group; ATV/r, atazanavir + ritonavir; DRV/r, darunavir + ritonavir; RAL, raltegravir. ^a^ The percentage of the modeled cohort remaining on treatment in the model was calculated based on the composite ACTG 5257 trial endpoint for discontinuation of the study drug for virologic or tolerability failure. An alternate trial endpoint was examined in scenario analysis.

ACTG 5257 clinical trial efficacy and tolerability data [2, 3] were used to transition and update the health and treatment status of the modeled cohort over the model time horizon. The model used the mean CD4 cell count increases from baseline to 24, 48, and 96 weeks observed during the trial to calculate transition probabilities between the CD4 cell count ranges (Fig 2A). The model first assumed that individuals started at the midpoint of their initial CD4 cell count range. Individuals were then shifted according to the mean CD4 cell count change from baseline and additionally were distributed according to the standard deviation of this change. These transition probabilities informed the distribution of individuals across the model health states at 24, 48, and 96 weeks.

In the model, individuals remained on their first-line ARV regimen until discontinuation due to virologic or tolerability failure (i.e., the primary composite endpoint of the ACTG 5257 clinical trial) after 24, 48, or 96 weeks of treatment (Fig 2B). Virologic failure was measured as confirmed HIV-1 RNA > 1,000 copies/mL at or after 16 weeks and before 24 weeks or > 200 copies/mL at or after 24 weeks. Tolerability failure was measured as discontinuation of the randomized regimen component for toxicity. ACTG 5257 trial results showed that virologic or tolerability failure by 96 weeks was significantly lower with the RAL regimen (8.6%) than with the other regimens (ATV/r: 24.1%; DRV/r: 16.6%) [2, 3]. An alternative clinical trial endpoint for discontinuation for any reason was tested in scenario analysis. Upon discontinuation of first-line ARV treatment (Fig 2B), individuals transitioned to a substitution regimen, based on the percentage of ACTG 5257 trial subjects who switched to that regimen (Table 2).

**Table 2:** Regimens used following discontinuation of randomized treatment in the ACTG 5257 clinical trial and associated antiretroviral drug costs.

Substitution Regimen^a^ [2, 3]	Initial Study Regimen			Cost per Day
	RAL	ATV/r	DRV/r	
RAL 400 mg BID + FTC/TDF 200/300 mg QD	0.0%	19.3%	48.3%	$98.57
ATV/r 300/100 mg QD + FTC/TDF 200/300 mg QD	28.1%	0.0%	24.1%	$109.14
DRV/r 800/100 mg QD + FTC/TDF 200/300 mg QD	40.6%	67.9%	0.0%	$109.65
Other regimens^b^ [3, 4]	31.3%	12.8%	27.6%	–
Total	100%	100%	100%	

ACTG, AIDS Clinical Trial Group; ATV/r, atazanavir + ritonavir; BID, twice daily; DRV/r, darunavir + ritonavir; EFV, efavirenz; FTC/TDF, emtricitabine/tenofovir disoproxil fumarate; QD, once daily; RAL, raltegravir; RPV, rilpivirine.

^a^ Individuals who discontinued their study regimen in the model were transitioned to various substitution regimens based on the percentage of participants who switched to each regimen in the trial.

^b^ Includes 12 alternate regimens (each used by five or fewer participants following discontinuation of any study regimen; the most common alternate regimens were EFV/FTC/TDF 600/200/300 mg QD and FTC/RPV/TDF 200/25/300 QD). The average costs per day of these other regimens were $109.47, $91.69, and $84.99 for participants discontinuing RAL, ATV/r, and DRV/r regimens, respectively.

#### Clinical inputs: Adverse event incidence

Grade 2, 3, or 4 adverse events with an incidence of at least 5% in any arm of the ACTG 5257 clinical trial were reported in Lennox et al. [2]). Corresponding grade 3 or 4 adverse events were included in the model to capture the events likely to be associated with substantive costs (Table 3).

**Table 3:** Adverse event incidence and management costs.

Adverse Event Category	Grade 3/4 Incidence^a^ [2]			Cost per Episode^b^ [5, 6]	Adverse Event Category from Cost Source^b^ [5]
	RAL	ATV/r	DRV/r		
Diarrhea	1.7%	1.8%	1.0%	$2,049	Diarrhea
Nausea	2.0%	1.5%	2.0%	$5,474	Nausea/vomiting
Vomiting	1.5%	1.3%	1.8%	$5,474	Nausea/vomiting
Abdominal pain	1.8%	3.0%	2.7%	$5,474	Nausea/vomiting
Headache	1.2%	2.0%	2.3%	$2,005	Headache
Pain in extremity	2.3%	2.5%	2.3%	$2,005	Headache^c^
Arthralgia	0.8%	1.3%	2.5%	$2,005	Headache^c^
Back pain	1.7%	0.7%	2.0%	$2,005	Headache^c^
Fatigue	0.8%	1.2%	1.2%	$3,407	Sleep-related symptoms
Elevated blood bilirubin level	0.8%	44.0%	0.7%	$1,925	Hepatoxicity

ATV/r, atazanavir + ritonavir; DRV/r, darunavir + ritonavir; RAL, raltegravir.

^a^ Grade 3/4 adverse event incidence values were taken from the ACTG 5257 clinical trial results (Table 3 of Lennox et al.) and were calculated as the number of individuals experiencing each event divided by the total number of individuals in each trial arm.

^b^ Cost estimates were taken from Simpson et al. and inflated from 2009 to 2016 US dollars. Adverse events without a cost estimate in Simpson et al. (cough, dyspnea, pyrexia, decreased blood phosphorus level, elevated blood glucose level) had similar incidence among trial arms and would therefore have minimal impact on incremental model results. Correspondingly, these adverse events were assumed to impose a $0 cost per episode.

^c^ Due to lack of more appropriate data, the model assumed that all reported types of grade 3/4 pain (except abdominal pain) would have management costs similar to grade 3/4 headache.

#### Costs

Individuals in the model incurred costs for ARV drug treatment, adverse event management, and HIV care. For each first-line ARV regimen, total daily ARV drug costs were calculated as the sum of the daily wholesale acquisition cost of each individual component of the regimen [4]. Resulting daily costs for each regimen were $98.57 for RAL, $109.14 for ATV/r, and $109.65 for DRV/r. For each substitution ARV regimen used by individuals who discontinued their first-line ARV regimen, total daily costs were also calculated by summing the wholesale acquisition cost of each individual regimen component. These substitution regimen costs were then weighted using the percentages of participants who switched to each regimen from their first-line ARV regimen (Table 2). The proportion of individuals on their first-line or substitution ARV regimen at baseline and at weeks 24, 48, and 96 were then used to calculate total ARV drug costs. Because discontinuations could occur between these time points, the model assigned the average cost between each pair of time points to the corresponding interval. For instance, ARV drug cost for the interval between weeks 24 and 48 was estimated as the average of the daily cost at week 24 and the daily cost at week 48, multiplied by the number of days in the 24-week time interval. This process was repeated for the baseline-to-24-week interval, the 24-to-48-week interval, and the 48-to-96-week interval. Total 96-week ARV drug costs for each arm of the model were calculated by summing across these three time intervals.

For adverse event management costs, the model applied a cost per episode to the percentage of individuals who experienced each adverse event. Because adverse events typically occur soon after treatment initiation, the model assumed that these costs were incurred within the first 48 weeks of treatment. The per-episode management cost associated with each adverse event was taken from Simpson et al. [5], which examined medical and pharmacy claims data for commercial and Medicare patients in the US during the 2004–2009 period and reported costs in 2009 US dollars. Similar costs for each adverse event category were reported in an alternative study by Dekoven et al. [7]. In the model, adverse event categories were appropriately mapped from Simpson et al. [5] to those chosen from the ACTG 5257 clinical trial for inclusion in the model (Table 3). Adverse events without a cost estimate in Simpson et al. [5] had similar incidence among trial arms and would therefore have minimal impact on the incremental model results. Correspondingly, these adverse events were assumed to impose a $0 cost per episode. Cost estimates obtained from Simpson et al. [5] were based on claims with a listed diagnosis corresponding to the adverse event; alternative (higher) adverse event costs provided in Simpson et al. [5] that were based on the incremental difference in overall medical and pharmacy costs between individuals with and without each adverse event were tested in scenario analysis. All cost estimates were inflated to 2016 US dollars [6]. Adverse event incidence and per-episode costs are summarized in Table 3.

Annual HIV care costs for disease monitoring and the treatment and prevention of opportunistic and other infections were taken from Gebo et al. [8] and included in the model. In this study, medical records were reviewed at 10 sites in the US HIV Research Network to assess resource utilization among adults with HIV-1 infection in primary HIV care in 2006. Resource use was multiplied by appropriate unit costs, and total HIV management expenditures were presented by cost category stratified across CD4 cell count ranges in 2006 US dollars. These costs were inflated to 2016 US dollars [6] and are summarized in Table 4. HIV care costs across the model time horizon were estimated by first multiplying the distribution of individuals within the CD4 cell count ranges in the model at baseline and at weeks 24, 48, and 96 by the HIV care costs associated with each CD4 cell count range. Then, because CD4 cell count changes could occur between these time points, average values were calculated for the baseline-to-24-week interval, the 24-to-48-week interval, and the 48-to-96-week interval, with a methodology similar to that used to estimate ARV drug costs. Total 96-week HIV care costs for each arm of the model were calculated by summing across these three time intervals.

**Table 4:** Average annual HIV care costs.

Cost Category^a^ [6, 8]	CD4 Cell Count Range (Cells/μL)^b^ [8]				
	< 50	51–200	201–350	351–500	> 500
N (Gebo et al. [8])	624	1,535	2,255	2,317	3,702
Inpatient costs	$25,344	$10,460	$4,555	$2,856	$2,234
Outpatient costs	$788	$855	$834	$794	$770
CD4 cell count test costs	$143	$161	$154	$152	$145
HIV-1 RNA test costs	$349	$389	$367	$364	$348
OI Px medication costs	$1,254	$801	$324	$194	$132
N (Gebo et al. [8])	226	623	945	1,034	1,430
Emergency department costs	$1,376	$621	$357	$263	$192
Non-HIV medication costs	$2,786	$2,705	$2,498	$2,542	$2,834
Total^c^	$32,039	$15,991	$9,090	$7,166	$6,654

OI, opportunistic infection; Px, prophylaxis.

^a^ Cost estimates were taken from Gebo et al. for available CD4 cell count ranges and were inflated from 2006 to 2016 US dollars. Not all sites contributed data for emergency department and non-HIV medication costs.

^b^ Individuals in the Gebo et al. study were assigned a CD4 cell count range based on their median CD4 cell count in 2006.

^c^ Discrepancies between total costs and column sums are due to rounding.

## Analyses

### Base-case analysis

Per-person 96-week costs, in total and by cost category, and the percentage successfully treated were estimated in the base-case analysis for each first-line ARV regimen. An annual rate of 3.0% was used to discount all cost outcomes [9].

### Scenario and sensitivity analyses

Scenario and sensitivity analyses were conducted to assess the robustness of the model results. In the scenario analysis, the total per-person cost was estimated for five additional scenarios: after 48 weeks of treatment; with first-line ARV treatment discontinuation for any reason; with no adverse event costs; with alternate adverse event costs [5]; and with no discounting of cost outcomes.

The joint impact of input parameter uncertainty on the model results was assessed in a probabilistic sensitivity analysis (PSA), in which all model input parameters for which there was sampling uncertainty were simultaneously varied according to the distributions summarized in S1 Table in the Supporting Information. Results from 10,000 Monte Carlo simulation runs were used to assess the distribution of costs, mean total costs, 95% confidence intervals, and the percentage of simulation runs in which each comparator had the lowest total costs.

## Results

### Base-case results

**Fig 3:**
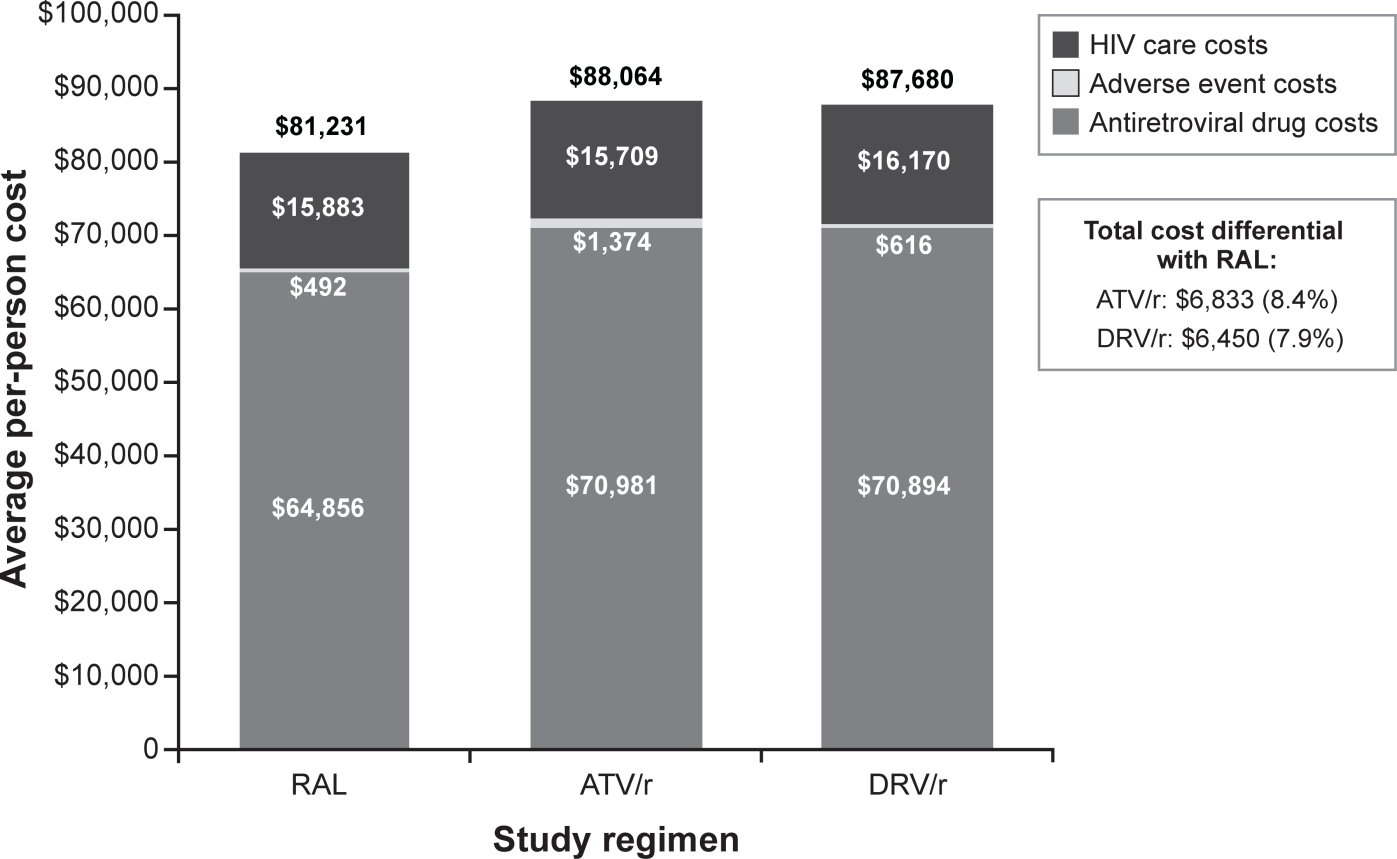
Base-case results: 96-week total costs. ATV/r, atazanavir + ritonavir; DRV/r, darunavir + ritonavir; RAL, raltegravir.

At 96 weeks, the cost analysis indicated that RAL was associated with lower ARV drug costs and adverse event costs, similar HIV care costs, and lower total costs when compared with ATV/r or DRV/r (Fig 3). Total costs were $81,231 for RAL, $88,064 for ATV/r, and $87,680 for DRV/r (Fig 3). A greater proportion of individuals receiving the RAL regimen was successfully treated (91.4%), according to the primary composite endpoint, compared with individuals receiving the ATV/r (75.9%) or DRV/r (83.4%) regimens.

### Scenario analysis results

The RAL regimen was found to have the lowest total per-person cost when compared with the ATV/r and DRV/r regimens in all scenarios tested (Table 5). The relative differences in costs between the RAL regimen and the ATV/r and DRV/r regimens in these scenarios were similar to the relative cost differences observed in the base-case results.

**Table 5:** Scenario analysis results.

Scenario	RAL	ATV/r	DRV/r
Base-case results^a^	$81,231	$88,064	$87,680
After 48 weeks of treatment	$41,993	$46,018	$45,498
With regimen switching based on discontinuation for any reason	$81,345	$88,087	$87,814
With no adverse event costs	$80,739	$86,690	$87,064
Using alternative adverse event costs [5]	$82,265	$90,657	$89,010
With no discounting of cost outcomes	$83,413	$90,403	$90,027

ATV/r, atazanavir + ritonavir; DRV/r, darunavir + ritonavir; RAL, raltegravir.

^a^ The base-case results represent the total per-person 96-week cost accounting for all modeled cost categories, using an annual discount rate of 3.0%. The modeled cohort was assumed to discontinue first-line treatment due to virologic or tolerability failure.

### Probabilistic sensitivity analysis results

**Fig 4:**
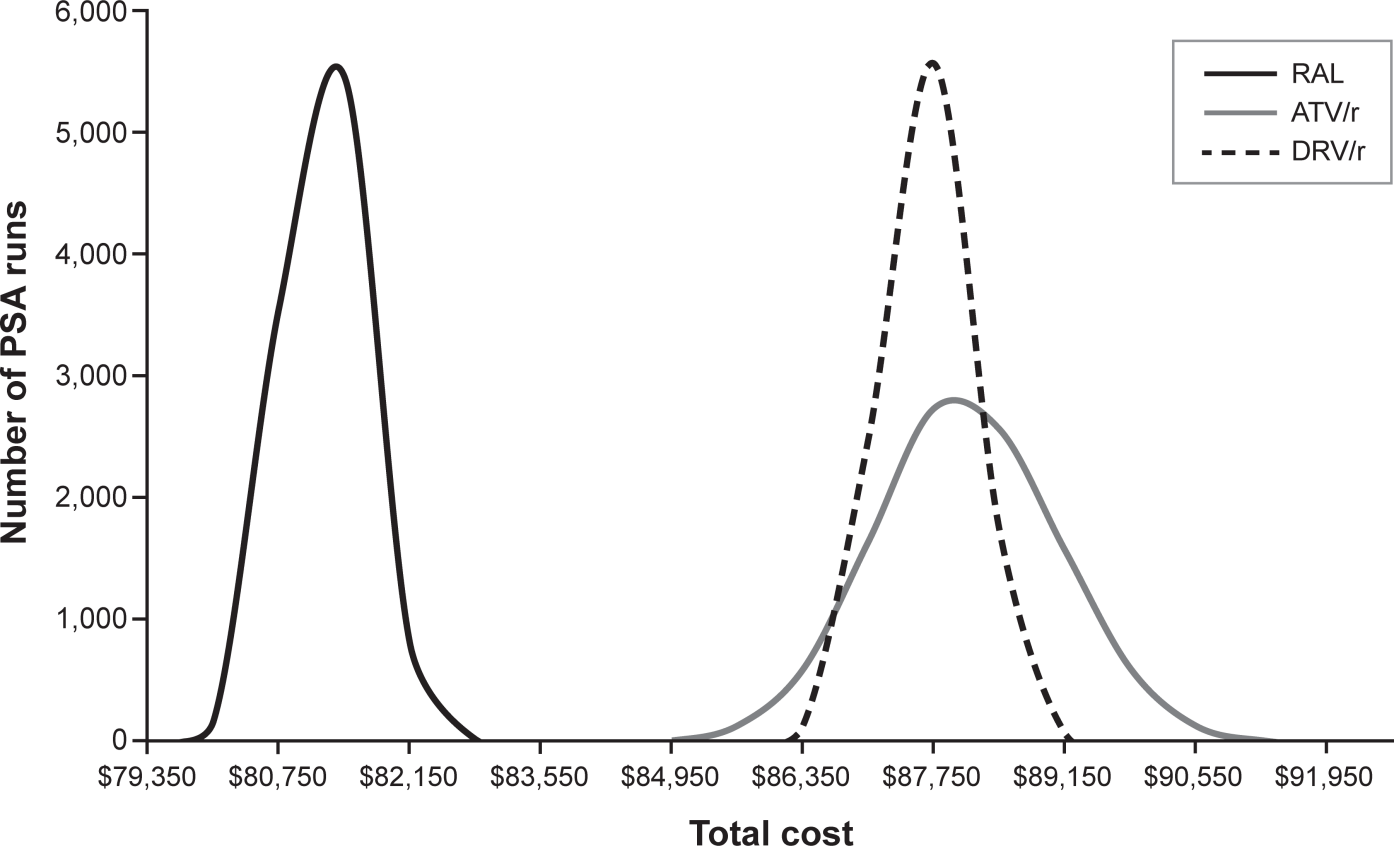
Distribution of probabilistic sensitivity analysis results. ATV/r, atazanavir + ritonavir; DRV/r, darunavir + ritonavir; PSA, probabilistic sensitivity analysis; RAL, raltegravir. Note: For ease of interpretation, this figure has been drawn as a continuous representation of a traditional histogram.

In all 10,000 simulations conducted for the PSA, RAL was found to have the lowest total per-person cost after 96 weeks of treatment (see S2 Table in the Supporting Information), yielding a distribution of PSA results that did not overlap with the distribution of results for either ATV/r or DRV/r (Fig 4). The mean total costs estimated by the PSA for each treatment arm were similar to those estimated in the base-case analysis (see S2 Table in the Supporting Information). ATV/r had the widest distribution in PSA results due to the increased incidence of adverse events (specifically elevated blood bilirubin level) among participants in this arm of the ACTG 5257 trial and the uncertainty around the associated costs per episode.

## Discussion

The objective of this study was to estimate the total HIV treatment costs among treatment-naive adults with HIV-1 infection in the US for three first-line integrase and protease inhibitors: RAL, DRV/r, and ATV/r, each used in combination with FTC/TDF. RAL, DRV/r, and ATV/r are recommended as initial ARV regimens in the US treatment guidelines [10] and were evaluated head-to-head in the ACTG 5257 clinical trial. Using efficacy, tolerability, and safety results directly from the ACTG 5257 clinical trial, our analysis found the RAL regimen to have the lowest per-person total cost after 96 weeks of treatment compared with the DRV/r and ATV/r regimens. Scenario and sensitivity analysis revealed the base-case results to be robust.

Our analysis is the first, to our knowledge, to directly compare these commonly used regimens in a US analysis, but several other studies provide context for our results. One previous study used ACTG 5257 head-to-head data to assess the economic impact of using DRV/r rather than ATV/r over 2 years in the United Kingdom [11]. As in our analysis, DRV/r was found to be cost saving relative to ATV/r, with average per-person cost savings of £393 over 2 years; however, this analysis excluded RAL and thus is not directly comparable with our findings. Another study used retrospective data from a US HIV cohort to compare the durability and cost of five ARV regimens, including RAL, ATV/r, and DRV/r, and similarly found RAL to be the most cost-effective option for treatment-naive individuals in a real-world clinical setting [12]. In an adaptation and expansion of our current economic model for Spain, RAL was found to be cost saving relative to DRV/r and ATV/r, with average per-person cost savings over 96 weeks of €1,550 and €4,251, respectively [13]. Other cost studies have included indirect comparisons and were primarily conducted for settings other than the US [14, 15] and therefore provide less relevant comparisons with our results. Future adaptations of our study beyond the US and Spain could help provide important additional information to decision makers.

This study is characterized by several strengths. The analysis relied on efficacy, tolerability, and safety data taken directly from ACTG 5257, a large head-to-head clinical trial that specifically studied first-line ARV regimens in a US population not treated with efavirenz. Second, the model had a 96-week time horizon and used only the data available from the ACTG 5257 clinical trial, thus avoiding the need for assumptions about long-term efficacy and treatment pathways. Finally, the robustness of the model’s results was examined and confirmed in scenario and sensitivity analyses.

Several limitations that are typical of pharmacoeconomic analyses should be considered when the cost-analysis results are interpreted. First, the analysis was limited to the three first-line ARV regimens studied in the ACTG 5257 clinical trial and did not include the once-daily formulation of RAL or other commonly used first-line ARV regimens for HIV treatment. The once-daily formulation of RAL has the same acquisition cost as the twice-daily version, with the potential for improved adherence, so we might expect slightly more favorable total cost results. This analysis should be extended to include other commonly used first-line ARV regimens if additional head-to-head clinical trial results become available. Second, because real-world adherence may be lower than in a clinical trial setting, HIV treatment costs observed in clinical practice may differ somewhat from those estimated in this analysis. In clinical practice, lower ARV drug costs (attributable to lower adherence) and higher HIV care costs (attributable to poorer health outcomes resulting from lower adherence) for all regimens may be observed. Further, this analysis estimated costs through 96 weeks of treatment and therefore did not capture potential long-term outcomes associated with RAL treatment, such as the benefits of RAL’s favorable lipid profile, as observed in the ACTG 5257 trial [2]. Finally, although data for adverse event costs and HIV care costs were somewhat dated, they were the most current estimates that could be identified.

## Conclusions

For treatment-naive adults with HIV-1 infection in the US, RAL + FTC/TDF had the lowest per-person total cost after 96 weeks of treatment when compared with DRV/r + FTC/TDF and ATV/r + FTC/TDF. This result was primarily due to lower ARV drug costs and adverse event costs for RAL + FTC/TDF than for the other two regimens. Scenario and sensitivity analyses revealed these findings to be robust. The results of this cost analysis complement the known clinical benefits of RAL [2, 3]. Further analysis should include additional geographic settings, other commonly used first-line ARV regimens, and the long-term outcomes of first-line RAL-based treatment for HIV-1 infection.

## Supporting information

S1 Table List of parameters tested in the probabilistic sensitivity analysis. PSA, probabilistic sensitivity analysis. ^a^ The Dirichlet distribution is the multivariate generalization of the beta distribution and can be used when there are more than two categories that must sum to 100%.
(DOCX)

S2 Table Probabilistic sensitivity analysis results. ATV/r, atazanavir + ritonavir; DRV/r, darunavir + ritonavir; RAL, raltegravir.
(DOCX)
