# Supplementary material for: Short-term cost analysis of raltegravir versus atazanavir + ritonavir or darunavir + ritonavir for treatment-naive adults with HIV-1 infection in the United States
Source: PLoS One. 2018 Aug 30;13(8):e0203293. doi: 10.1371/journal.pone.0203293 (PMC6117059; doi:10.1371/journal.pone.0203293)
Supplement: S1 Table — PSA, probabilistic sensitivity analysis. a The Dirichlet distribution is the multivariate generalization of the beta distribution and can be used when there are more than two categories that must sum to 100%. (DOCX) [file pone.0203293.s001.docx]

1. List of Parameters Tested in the Probabilistic Sensitivity Analysis.

| Input Parameter | Base Case | Distribution for PSA |
| --- | --- | --- |
| Baseline CD4 cell count distribution | Pooled distribution from Lennox et al. (2014) | Dirichlet^a^ |
| Mean change in CD4 cell count from baseline | Regimen- and week-specific changes from Lennox et al. (2014) | Normal |
| Cumulative percentage experiencing treatment discontinuation over time | Regimen- and week-specific percentages from Lennox et al. (2014) | Beta |
| Percentage of participants switching to each substitution regimen following discontinuation of randomized treatment | Regimen-specific percentages from Ribaudo et al. (2013) | Dirichlet^a^ |
| Adverse event incidence | Regimen-specific incidence from Lennox et al. (2014) | Beta |
| Adverse event management costs | Simpson et al. (2014) | Normal |
| Mean annual cost of HIV care by CD4 cell count range | Gebo et al. (2010) | Normal |

PSA = probabilistic sensitivity analysis.

^a^ The Dirichlet distribution is the multivariate generalization of the beta distribution and can be used when there are more than two categories that must sum to 100%.
