# Supplementary material for: Short-term cost analysis of raltegravir versus atazanavir + ritonavir or darunavir + ritonavir for treatment-naive adults with HIV-1 infection in the United States
Source: PLoS One. 2018 Aug 30;13(8):e0203293. doi: 10.1371/journal.pone.0203293 (PMC6117059; doi:10.1371/journal.pone.0203293)
Supplement: S2 Table — ATV/r, atazanavir + ritonavir; DRV/r, darunavir + ritonavir; RAL, raltegravir. (DOCX) [file pone.0203293.s002.docx]

1. Probabilistic Sensitivity Analysis Results.

| Regimen | Base-Case Total Cost | Probabilistic Sensitivity Analysis Results | | |
| --- | --- | --- | --- | --- |
|  |  | Percentage of Runs With the Lowest Cost | Mean Total Cost | 95% Confidence Interval |
| RAL | $81,231 | 100% | $81,234 | $80,444 – $82,024 |
| ATV/r | $88,064 | 0% | $88,088 | $86,195 – $89,982 |
| DRV/r | $87,680 | 0% | $87,686 | $86,803 – $88,568 |

ATV/r = atazanavir + ritonavir; DRV/r = darunavir + ritonavir; RAL = raltegravir.
